# Effects of humorous interventions on the willingness to donate organs: a quasi-experimental study in the context of medical cabaret

**DOI:** 10.1186/s12889-020-8400-y

**Published:** 2020-03-04

**Authors:** Lisa Heitland, Eckart von Hirschhausen, Florian Fischer

**Affiliations:** 1 Bielefeld University, School of Public Health, P.O. Box 100 131, 33501 Bielefeld, Germany; 2 Foundation “Humor Hilft Heilen”, Bennauer Str. 31, 53115 Bonn, Germany

**Keywords:** Health communication, Medical cabaret, Humor, Organ donation, Organ transplantation

## Abstract

**Background:**

It has been shown that fears and misconceptions negatively affect the willingness to donate organs. Empirical studies have examined health communication strategies that serve to debunk these fears. There are promising indications that humor has the potential to influence health-related attitudes and behaviors. This study examines empirically whether medical cabaret, as a specific format for delivering health-related information in a humorous way, affects the willingness to donate organs.

**Methods:**

A quasi-experimental study was conducted among the audience of a medical cabaret live show. Participants in two intervention groups and one control group were interviewed just before the start of the live show (t_0_) and about 6 weeks later (t_1_). Intervention group 1 (I_1_) witnessed a ten-minute sequence by the cabaret artist about organ donation. Participants in I_2_ witnessed the sequence and, in addition, received an organ donor card. Descriptive statistics and t-tests were used to investigate changes in attitudes and the willingness to donate organs from t_0_ to t_1_.

**Results:**

A significant increase in the willingness to donate organs and an improvement in general attitude was observed in the intervention groups. Moreover, significantly more participants in I_2_ carried an organ donor card after the intervention. Some fears could be reduced, while understanding of the reasons for organ donation could be increased via the intervention.

**Conclusions:**

The study confirms that medical cabaret is able to affect respondents’ attitudes and behaviors even in the context of organ donation. Medical cabaret can enhance the willingness to donate organs and dispel negative concerns.

## Background

The mismatch between the number of donor organs required and those available poses a major challenge to the Eurotransplant region. Currently, there are 14,129 people waiting for a donor organ [1], and many of them will die while on the waiting list for an organ transplant. Different regulations in terms of opt-in or opt-out solutions regarding organ donation exist. For example, in Germany organs can only be taken from people who have declared their willingness by carrying around an organ donor card (opt-in solution). Declining rates of organ transplantation have led to discussions about introducing a law that would automatically make everyone a registered organ donor but give them the right to opt out. An additional hurdle should be that relatives can refuse organ transplantation from a loved one after death, so that this approach is now called the “dual opt-out” solution.

In order to increase the number of organ donors, it is necessary to identify the fears and factors related to the (lack of) willingness to donate organs. Although the general attitude towards organ donation is a significant predictor of individual willingness [2], the reported correlation coefficient between attitude and willingness related to organ donation is relatively small [3]. Therefore, it is necessary to differentiate the determinants of willingness to donate organs. Parisi and Katz [4] were able to prove that attitude is not a one-dimensional construct, but that it should be measured using two subscales, prodonation and antidonation. The prodonation scale emphasizes the humanitarian benefits of donating organs, as well as personal feelings such as contentment and pride, and thus identifies reasons for organ donation. Factors that favor readiness for postmortem organ donation include altruistic ideas [2]. People who agree to postmortem organ donation often cite the reason of helping others with their own deaths through organ donation [5–7]. In addition, social influences are conducive to people feeling ready for organ donation [2].

The personal fears that prevent people from donating organs are among the factors on the antidonation scale, according to Parisi and Katz [4]. This negative dimension includes, for example, the fear of physical mutilation [5, 8–10] and the fear of inadequate medical care [4, 5, 9]. People who reject organ donation often doubt that irreversible brain function failure genuinely proves death in humans [11–14]. There is also evidence that general anxiety or discomfort before one’s death may also be a reason for rejecting organ donation [2, 15, 16]. Frequently, these fears are associated with distrust of the physicians and institutions involved in transplantation medicine [11, 17].

In order to achieve higher numbers of organ donations, it is important to establish and evaluate effective measures to promote willingness to donate. According to systematic reviews, the focus of interventions aimed at promoting organ donation readiness should be to reduce anxiety [2, 18].

### Humor in health communication

Although humor is frequently used in advertisements, it is not yet an established tool in the communication of health-related issues. However, there are initial indications that humor can be an effective strategy in health communication [19–22]. Studies have shown that humor significantly boosts an audience’s attention and allows them to recall the content later [19, 21]. Humorous messages in the field of health communication can continue to help recipients communicate with other people about the mediated topic and, thereby, to disseminate the topic [23]. Humor seems to have an effect even in cases when the recipient has previously had a negative attitude towards the topic being addressed [20]. Humorous messages can evoke positive, affective feelings that have the potential to reduce anxiety and excess tension [20, 24]. In particular, the combination of fear and humor can increase the persuasive effects of arguments relating to health issues [25]. In terms of willingness to donate organs, there are some promising initial empirical findings demonstrating that a humorous tone is more effective than a sad one when motivating individuals to donate [26, 27]. However, as yet, humor has rarely been used as a communication tool for health-related issues [28, 29].

A special format for humor in health communication is medical cabaret. It has only recently gained attention, because health communication and medical cabaret pursue a common goal: the content of the messages should inspire the audience or the target group to reflect and subsequently achieve change in the best possible way [29]. The medical cabaret artist (usually) has a medical education or background in the medical field and aims to entertain his or her audience through various elements, such as text, music, film, and other elements [29, 30]. While people often pick things up in an unfocused way in everyday life, the audience of a medical cabaret is exposed to the given topic for a longer time without any disruptive effects [29]. The effectiveness of medical cabaret on attitudinal and behavioral changes was first tested in the context of the topic of school and alternative medicine [31]. This study has shown that medical cabaret can change health-related attitudes.

### Aim

Against the background of these findings, the study presented here aimed to measure the effects of medical cabaret in the context of organ donation. In order to do so, a sequence addressing the topic of organ donation within a live show of medical cabaret (“Endlich!” [“Finally!”] by the most famous German medical cabaret artist, Dr. Eckart von Hirschhausen) was used as an intervention to evaluate its impact on willingness to donate organs. This study illustrates the main predictors of organ donation in terms of psychological aspects. It addresses the following questions related to the impact of humor in the area of organ donation: What are the effects of a humorous intervention in the form of medical cabaret on:

- general attitude towards organ donation,
- individual willingness to donate organs,
- possession of an organ donor card, and
- predictors of organ donation (prodonation and antidonation)?

## Method

### Design

A quasi-experimental study among the audiences of three live show events of medical cabaret was conducted. The study design included a pre- and post-survey, with one control group and two intervention groups, to examine the effect of medical cabaret on attitudes towards organ donation and the willingness to donate.

### Participants, procedure, and intervention

The audiences from three live shows of medical cabaret by Dr. Eckart von Hirschhausen received a paper-and-pencil-based standardized questionnaire directly before the beginning of the live show (t_0_). About 6 weeks after the live show, the respondents were contacted again to take part in the post-survey (t_1_), via either an online-based or a paper-and-pencil-based questionnaire, depending on the preferences of respondents. An individualized code enabled us to merge data from t_0_ and t_1_.

**Fig. 1:**
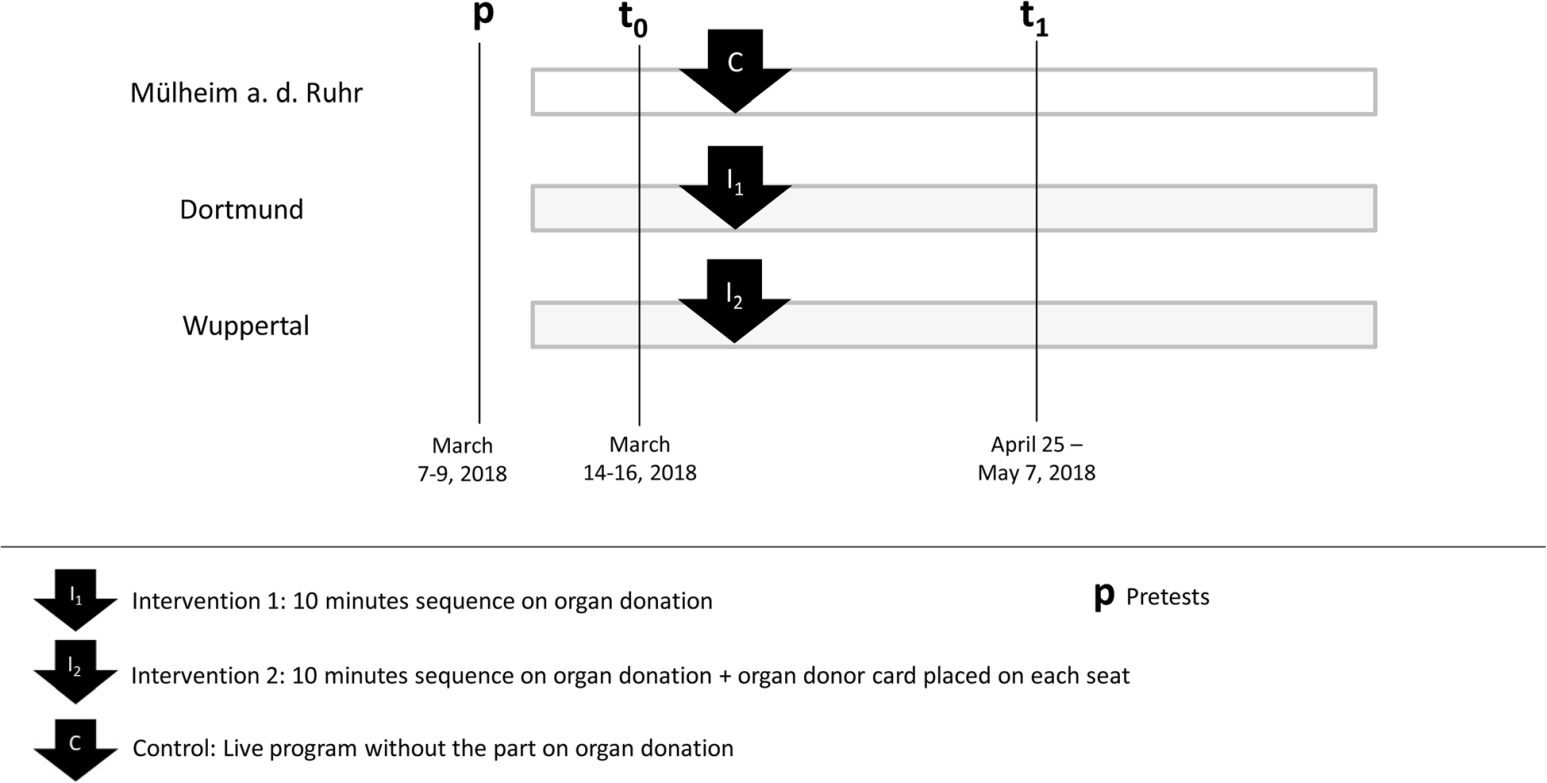
Framework of the study design and data collection

The audiences at all three live shows were deemed to be comparable, because all three locations were close to each other in the Ruhr area of Germany (Mülheim a.d. Ruhr, Wuppertal, and Dortmund) and data collection for t_0_ took place on three consecutive evenings in March 2018. The control group (C) witnessed the usual program of the live show “Endlich!”, while the first intervention group (I_1_) witnessed the usual program plus an additional 10 min lasting sequence on the subject of organ donation. The second intervention group (I_2_) witnessed the same sequence on organ donation and, furthermore, organ donor cards were placed on each seat (Fig. 1).

Within the intervention, the present situation of a declining number of organ donors in Germany was introduced. Humorous messages were used to point out that most of the donor organs transplanted into German patients come from abroad by putting this into the context of industrial import and export. It was explicitly stated that the lack of donor organs in countries with an opt-in solution is a consequence of the discrepancy between attitudes and behavior and that several fears lead to the avoidance of a positive decision regarding organ donation. The intervention sensitized the audience to the irrational fears related to organ donation and used mechanisms to debunk all of these fears in a humorous manner. By the end of the sequence, the audience’s empathy should be awakened, through appeals to humanity, solidarity and generosity.

### Measures

The survey instrument was a standardized questionnaire based on theoretical considerations and results from previous empirical studies related to fears about organ donation (antidonation) and reasons for organ donation (prodonation). As far as possible, validated instruments were used or adapted. The whole questionnaire underwent a qualitative and quantitative pretest. The sociodemographic characteristics of age (in years) and sex (“female”, “male”, and “other”) were assessed.

#### Attitudes

The general attitude was operationalized by the question: “What is your general opinion regarding organ donation?” This could be answered by the participants with “rather positive”, “neutral”, or “rather negative”. In addition, the participants were also given the opportunity to answer: “I have not yet considered the topic”.

#### Willingness to donate organs

The items relating to concrete willingness to donate organs are based on the options that are offered on an organ donor card in Germany. The participants were able to tick, first, whether they have an organ donor card or not. Participants who have an organ donor card could then tick whether they have agreed to an organ donation, rejected it, or transferred the decision to another person after their death. People who do not have an organ donor card could choose between the following answer options: “No, but I intend to get one soon”, “No, but I would agree to an organ donation”, “No, and I would not agree to an organ donation”, and “No, because I am not eligible to donate organs for medical reasons”. For undecided participants, there was also the answer: “I have not yet made a decision”. In this study, willingness to donate was assumed if: 1) the participants had an organ donor card and stated that they had agreed to donate organs or 2) if participants did not have an organ donor card but indicated that they consented to donating organs.

#### Psychological factors

The assessment of psychological factors relates to beliefs and fears, containing questionnaires on reasons for (prodonation; Table 2) or against (antidonation; Table 3) an organ donation. The personal reasons related to prodonation were assessed via five items, using a six-point Likert scale (“strongly disagree”, “disagree”, “tend to disagree”, “tend to agree”, “agree”, and “fully agree”). The antidonation scale, which should reflect the reservations and fears of the audience, included eight items with the same six-point Likert scale.

#### Additional items (t_1_)

In addition to the items described above, another ten items were added to the questionnaire at t_1_. In order to be able to take into account possible external influences on willingness to donate organs, the participants were asked whether they had actively looked for information on organ donation in the past 6 weeks or whether they had come into contact with the issue of organ donation in other ways. Furthermore, participants were asked to provide an appraisal of how much they liked the live show “Endlich!” overall on a scale from 1 to 10. The participants in the intervention groups were also asked to give an assessment of the sub-aspect of organ donation. For this, they could choose whether they appraised this part as “much less entertaining”, “less entertaining”, “just as entertaining”, “more entertaining” or “much more entertaining” than the other parts of the live show. On further six-point Likert scales, the participants in the follow-up survey were also asked to express their agreement as to whether the live show had led them to: form their own opinion regarding organ donation; obtain further information about organ donation; talk with relatives and/or friends about organ donation; or complete an organ donor card or make changes to it.

### Data analysis

Socio-demographic data, information on general attitudes towards organ donation and organ donation readiness, as well as the answers relating to prodonation and antidonation, were analyzed descriptively on the basis of the t_0_-values.

In order to calculate the impact of individual psychological items of factors related to prodonation and antidonation on the willingness to donate, simple logistic regression models were calculated. The requirements were checked in advance. Here, there was a strong multicollinearity between the variables of the prodonation scale and also between the variables of the antidonation scale. Each reason for or against organ donation was individually included in a regression model for describing how individual attitudes and fears affect the likelihood of whether a study participant agrees to post-mortem organ donation or not. Thus, first conclusions can be drawn about the direction and strength of the relationship between the individual items and willingness to donate. A direct interpretation of the regression coefficients should be avoided.

In order to investigate the overall effects of factors related to prodonation and antidonation on willingness to donate, sum indices were calculated. Beforehand, reliability analyzes were conducted. Sum indices were formed, taking into account internal consistency based on Cronbach’s alpha. Due to the multicollinearity, the two scales were not used in a common regression equation.

A non-responder analysis was performed to investigate whether the persons participating in both the pre- and post-survey differed from those who only participated in the pre-survey. The nonresponse analysis included the following variables: age, gender, general attitude towards organ donation and willingness to donate, and possession of an organ donor card. The non-responder analysis showed that younger or middle-aged female individuals with a more positive attitude to organ donation and those who already had organ donor cards and/or were prepared to donate organs were more likely to participate in both the pre- and post-survey.

The effects of medical cabaret on willingness to donate organs were determined using the pre- and post-survey data. The individual prodonation and antidonation items were checked for changes between the before and after show judgements for each group. Due to the interval-scaled measurement level of the variables, t-tests for dependent samples were suitable. Cohen’s d was calculated using a correction factor to calculate the effect sizes. The respective correction factor is based on the correlation between pre- and post-measurement as well as the standard deviation of the pretest value [32]. The Wilcoxon signed-rank test was used for the non-parametric variable assessing the overall attitude towards organ donation. The McNemar test was used for the variable related to the possession of an organ donor card and the variable that operationalizes willingness to donate organs. Differences between t_0_ and t_1_ measurements were found to be significant at a level of *p* < 0.05. Significant results were considered to be evidence that the medical cabaret did have an effect on the variables in question.

## Results

### Sample characteristics

**Fig. 2:**
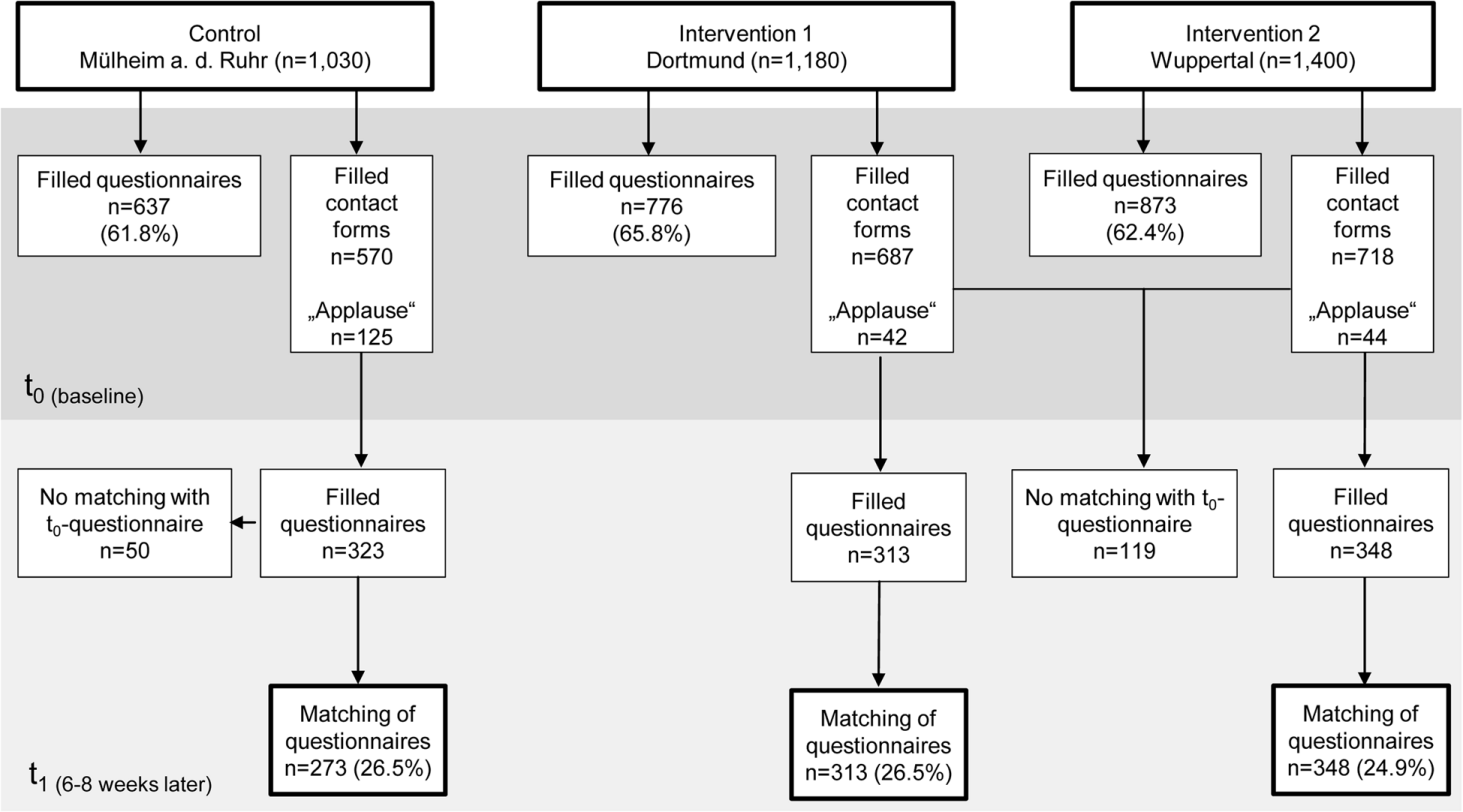
Flow chart for recruitment

The audiences for the three live shows varied between 1030 and 1400 people. A total of 2286 people participated in the pre-survey. The response rate for the three groups in the t_0_-survey was between 61.8 and 65.8%. In the post-survey, a total of 1103 people completed the questionnaire (t_1_). Of these, 934 questionnaires (I_1_: 313; I_2_: 348; C: 273) could be matched with the t_0_-questionnaires. Therefore, the response rates were between 24.9 and 26.5% (Fig. 2).

The mean age of the entire sample at t_0_ was 47.8 years (*n* = 2204; SD = 15.5). The subjects in the control group were aged between 15 and 83 years (M = 46.2; SD = 15.8), in I_1_ between 14 and 86 years (M = 46.7; SD = 14.9) and in I_2_ between 12 and 92 years (M = 49.9; SD = 15.5). Overall, slightly more than 60% of the study participants at t_0_ were female in all three groups (Table 1).

**Table 1:** Sample characteristics at baseline

	I_**1**_ (***n*** = 776)		I_**2**_ (***n*** = 873)		C (***n*** = 637)	
	***n***	%	***n***	%	***n***	%
**Sex**						
Male	300	39.4	313	37.4	246	39.4
Female	462	60.6	524	62.6	378	60.6
**Age (in years)**						
≤ 25	58	7.5	66	7.6	61	9.6
26–35	152	19.6	111	12.7	134	21.1
36–45	122	15.7	100	11.5	75	11.8
46–55	204	26.3	242	27.7	174	27.4
56–65	133	17.1	190	21.8	96	15.1
66–75	62	8.0	85	9.7	61	9.6
≥ 76	45	5.8	79	9.0	33	5.2

Values may not sum up to the total amount due to missing values

### Attitudes towards organ donation

Between 64 and 68% of respondents stated at t_0_ that they had a positive attitude towards organ donation. About 20% of respondents indicated a neutral attitude. At t_0_, between 4 and 6% had not yet dealt with the topic and about 6 to 8% of the respondents stated a rather negative attitude towards organ donation. Within the overall sample, 41.8% (*n* = 932) of the study participants had an organ donor card prior to the live show. In total, 1029 persons (around 45% of the total sample at time t_0_), regardless of whether or not they had an organ donor card, stated that they would consent to organ donation (I_1_: 48.5%; I_2_: 42.6%; C: 48.1%). Of these persons, 65.6% had documented their willingness with an organ donor card. The remaining 354 participants (34.4%) agreed in principle to organ donation, but had not yet documented this with an organ donor card.

### Predictors of willingness to donate organs

**Fig. 3:**
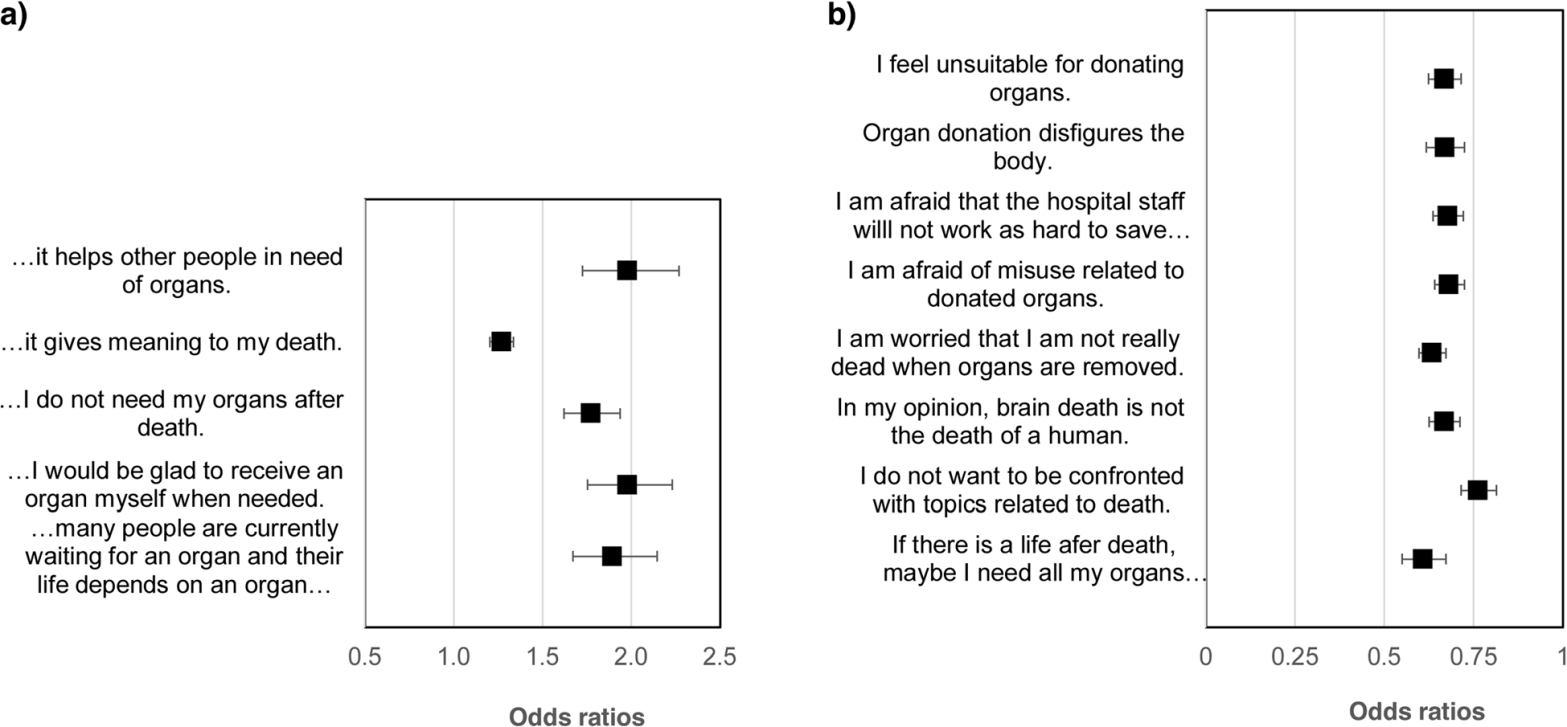
Predictors of willingness to donate organs in terms of prodonation (**a**) and antidonation (**b**)

Age showed a significant, but very slight, effect on willingness to donate (OR = 0.985, 95% CI: 0.980–0.991, *p* < 0.001). Gender does not significantly affect willingness (OR = 0.882, 95% CI: 0.743–1.047, *p* = 0.151). The coefficients of all the variables related to both prodonation and antidonation remained highly significant even after adjustment for age, and the odds ratios changed only slightly. For this reason, the unadjusted odds ratios are presented in the following forest plots (Fig. 3).

All prodonation statements have a single, isolated, highly significant effect on willingness to donate (*p* < 0.001). It can be stated that the impact of altruistic ideas (OR = 1.978, 95% CI: 1.724–2.269, *p* < 0.001) and reciprocity (OR = 1.977, 95% CI: 1.754–2.230, *p* < 0.001) are most prominent (Fig. 3a).

As the second forest plot (Fig. 3b) shows, all variables on the antidonation side were confirmed to be highly significant predictors of willingness to donate (*p* < 0.001). A greater approval of these variables reduces the likelihood that a study participant will consent to organ donation. The more they agree with the statements about reservations and fears, the less likely they are to donate organs after death. The fear that organs might be needed postmortem in case there is a life after death has the most pronounced negative impact on willingness to donate (OR = 0.609, 95% CI: 0.551–0.673, *p* < 0.001). The other odds ratios are between 0.634 and 0.763 and, thus, close to each other.

Attitudes towards organ donation are not a one-dimensional construct. The humanitarian benefits of organ donation were summarized by Parisi and Katz [4] in a prodonation scale. The antidonation scale, on the other hand, includes the negative dimensions of organ donation. In order to analyze the extent to which these two dimensions affect the outcome of willingness to donate organs, we used these two subscales as well. To determine the internal consistency, Cronbach’s alpha was calculated for both scales. Internal consistency improves when omitting the item “Organ donation is meaningful, because it gives meaning to my death”. Thus, the prodonation scale was formed based on the remaining variables. The scale ultimately reached a Cronbach’s alpha of 0.846 for t_0_ and 0.853 for t_1_. The antidonation scale (excluding the item “I feel unsuitable for donating organs”) achieved an internal consistency of 0.769 for t_0_ and 0.825 for t_1_.

Both sum indices for the prodonation and antidonation scale are significantly associated with willingness to donate. If approval on the prodonation scale increases by one unit, the relative probability of readiness to donate increases by about 30% (OR = 1.301, 95% CI: 1.248–1.356, *p* < 0.001). If the model consists solely of the prodonation scale, Nagelkerke’s R^2^ is 0.141. According to Cohen (1992), this corresponds to a strong effect on willingness to donate organs (f = 0.41). In a binary logistic regression model, in which only the antidonation scale is included as an independent variable, the Nagelkerke’s R^2^ is higher. It is 0.201 and, therefore, also has a strong effect on willingness to donate (f = 0.50). Reducing consent on the antidonation scale significantly reduces the relative likelihood of willingness to donate (OR = 0.874, 95% CI: 0.859–0.888, *p* < 0.001).

### Willingness to donate organs and the live show

The live show was very well liked by the majority of study participants. On a scale of 1 to 10, 87.6% of viewers rated it as 7 or better. The mean was 8.2 (SD = 1.9). However, 28.9% (*n* = 190) of respondents in the intervention groups judged the organ donation sequence to be less entertaining than the other content of the live show.

At t_1_, 63.0% of the intervention groups stated that the live show had increased their interest in organ donation (*n* = 415). At t_1_, 5.7% (*n* = 52) said they actively searched for more information on organ donation after the live show and 189 persons (20.3%) came into contact with the issue of organ donation in another way after the live show. Almost half of the participants in the intervention groups (45.6%; *n* = 301) claimed that the live show had provided them with the opportunity to form their own opinion regarding organ donation. In addition, a large proportion of the intervention groups (60.4%; *n* = 399) agreed that the show had moved them to talk to relatives and/or friends about organ donation. In addition, 203 people out of 659 participants in the intervention groups (30.8%) agreed that the live show had demonstrated to them that society expects them to complete an organ donor card.

### Changes in terms of attitudes

In relation to all the study participants for whom a completed questionnaire was available for both the pre- and post-surveys, 78 respondents (8.5%) showed a positive change in their general attitude towards organ donation. These individuals reported a better overall attitude towards organ donation 6 to 8 weeks after the live show compared to before they had seen it. These changes were significant only in the intervention groups (I_1_: *p* = 0.003; I_2_: *p* = 0.004) and not in the control group (*p* = 0.835). Focusing on changes in attitudes among those persons who stated in the pre-survey that they had a neutral or negative attitude, even more pronounced effects are visible: In 33.2% (*n* = 75) of these individuals a slight change, and in 1.3% (*n* = 3) even a strong change, in the overall attitude has been achieved.

### Changes in willingness to donate organs

Willingness to donate organs increased significantly in I_1_ (*p* < 0.001): While 56.2% (*n* = 176) of the study participants were willing to donate organs before the intervention, 66.8% (*n* = 209) were willing to do so afterwards. In I_2_ as well, the readiness to donate changed significantly (*p* < 0,001): While 53.8% (*n* = 184) of study participants expressed their willingness to donate organs before the live show intervention, this had risen to 66.3% (*n* = 230) 6 weeks after the intervention.

In addition to willingness to donate organs, it was further investigated whether there was an increase in the number of those who had an organ donor card after the interventions. The number of organ donors had increased in both intervention groups. In I_1_, 11 respondents who did not previously have an organ donor card had one after the live show. However, the number of study participants with an organ donor card did not differ significantly from t_0_ to t_1_ (*p* = 0.057). In I_2_, in which organ donor cards were distributed at the live show, however, a significant increase in the number of organ donor card possessors could be seen (*p* = 0.001). Before the live show, 58.2% (*n* = 199) of respondents in this group stated that they had an organ donor card. In the follow-up survey, the proportion of these persons had increased to 62.8% (*n* = 218). No significant changes were seen in the control group, where a slight decrease in organ donor card possession was in fact observed.

### Changes in predictors of willingness to donate organs

Comparisons of the individual mean values ​​of the prodonation and antidonation variables before and after the experiment were performed. A positive mean difference in prodonation indicates a more positive attitude (Table 2). With regard to the antidonation variables, negative values of the mean differences indicate a greater rejection of the reservations and fears (Table 3).

**Table 2:** Changes in prodonation items and scale from t_0_ to t_1_

	Organ donation is meaningful, because...	t_**0**_		t_**1**_		Parametric tests			
		M	SD	M	SD	M_**diff**_	t	***p***-value^**a**^	Cohen’s d
I_1_	… it helps other people in need of organs.	5.70	0.83	5.71	0.63	0.01	−0.25	0.805	0.01
	… it gives meaning to my death.	3.44	1.64	3.70	1.58	0.26	−3.29	0.001	0.19
	… I do not need my organs after death.	5.02	1.23	5.29	0.99	0.27	−3.95	< 0.001	0.21
	… I would be glad to receive an organ myself when needed.	5.52	0.96	5.63	0.76	0.11	−1.83	0.068	0.10
	… many people are currently waiting for an organ and their life depends on an organ transplantation.	5.58	0.89	5.57	0.70	−0.01	0.17	0.863	−0.01
	*Prodonation scale*	*21.84*	*3.23*	*22.19*	*2.45*	*−0.35*	*0.02*	*0.065*	*0.10*
I_2_	… it helps other people in need of organs.	5.69	0.71	5.58	0.87	−0.11	2.40	0.017	−0.15
	… it gives meaning to my death.	3.43	1.76	3.61	1.67	0.18	−2.21	0.028	0.12
	… I do not need my organs after death.	5.28	1.05	5.34	0.99	0.06	−1.25	0.214	0.06
	… I would be glad to receive an organ myself when needed.	5.56	0.87	5.46	0.99	−0.10	2.11	0.036	−0.13
	… many people are currently waiting for an organ and their life depends on an organ transplantation.	5.59	0.73	5.52	0.84	−0.07	1.58	0.116	−0.09
	*Prodonation scale*	*22.21*	*2.64*	*21.97*	*3.18*	*0.24*	*1.60*	*0.110*	*−0.10*
C	… it helps other people in need of organs.	5.66	0.80	5.64	0.689	−0.02	0.43	0.669	−0.02
	… it gives meaning to my death.	3.49	1.64	3.66	1.559	0.18	−1.90	0.059	0.11
	… I do not need my organs after death.	5.23	1.17	5.26	0.948	0.03	−0.49	0.622	0.03
	… I would be glad to receive an organ myself when needed.	5.56	0.92	5.58	0.779	0.02	−0.39	0.694	0.02
	… many people are currently waiting for an organ and their life depends on an organ transplantation.	5.62	0.85	5.51	0.726	−0.11	1.97	0.050	−0.11
	*Prodonation scale*	*22.14*	*3.11*	*22.00*	*2.44*	*0.14*	*0.79*	*0.431*	*−0.04*

^a^ *p*-values based on t-test for paired samples

**Table 3:** Changes in antidonation items and scale from t_0_ to t_1_

	Fears and caveats	t_**0**_		t_**1**_		Parametric tests			
		M	SD	M	SD	M_**diff**_	t	p-value^**a**^	Cohen’s d
I_1_	I feel unsuitable for donating organs.	1.98	1.21	2.18	1.30	0.19	−2.94	0.003	0.18
	Organ donation disfigures the body.	1.93	1.11	1.97	1.09	0.05	−0.86	0.393	0.04
	I am afraid that the hospital staff will not work as hard to save my life when I have an organ donor card.	2.51	1.40	2.39	1.32	−0.12	1.94	0.053	−0.11
	I am afraid of misuse related to donated organs.	2.88	1.45	2.71	1.30	−0.18	2.79	0.006	−0.15
	I am worried that I am not really dead when organs are removed.	2.76	1.62	2.40	1.42	−0.35	5.42	< 0.001	−0.30
	In my opinion, brain death is not the death of a human.	2.38	1.36	2.22	1.34	−0.16	2.08	0.039	−0.13
	I do not want to be confronted with topics related to death.	2.05	1.21	2.19	1.19	0.15	−2.27	0.024	0.12
	If there is a life after death, maybe I need all my organs for it.	1.47	0.82	1.47	0.75	0.00	0.11	0.915	0.00
	*Antidonation scale*	*15.91*	*6.01*	*15.31*	*5.74*	*0.60*	*2.73*	*0.007*	*−0.16*
I_2_	I feel unsuitable for donating organs.	2.13	1.43	2.20	1.40	0.07	−1.03	0.304	0.06
	Organ donation disfigures the body.	1.82	1.19	1.93	1.14	0.11	−2.06	0.040	0.11
	I am afraid that the hospital staff will not work as hard to save my life when I have an organ donor card.	2.32	1.41	2.23	1.28	−0.09	1.60	0.110	−0.08
	I am afraid of misuse related to donated organs.	2.88	1.47	2.76	1.39	−0.12	1.95	0.052	−0.10
	I am worried that I am not really dead when organs are removed.	2.60	1.61	2.38	1.44	−0.22	3.96	< 0.001	−0.20
	In my opinion, brain death is not the death of a human.	2.27	1.46	2.13	1.29	−0.14	1.90	0.058	−0.10
	I do not want to be confronted with topics related to death.	2.03	1.30	2.23	1.32	0.20	−3.31	0.001	0.18
	If there is a life after death, maybe I need all my organs for it.	1.52	0.98	1.49	0.90	−0.03	0.47	0.639	− 0.03
	*Antidonation scale*	*15.31*	*6.61*	*15.08*	*6.39*	*0.23*	*1.01*	*0.315*	*−0.06*
C	I feel unsuitable for donating organs.	1.96	1.28	2.14	1.30	0.18	−2.88	0.004	0.18
	Organ donation disfigures the body.	1.73	1.05	1.94	1.10	0.21	−3.61	< 0.001	0.22
	I am afraid that the hospital staff will not work as hard to save my life when I have an organ donor card.	2.55	1.47	2.51	1.38	−0.04	0.54	0.587	−0.04
	I am afraid of misuse related to donated organs.	3.01	1.48	2.94	1.41	−0.07	1.03	0.304	−0.06
	I am worried that I am not really dead when organs are removed.	2.83	1.63	2.74	1.44	−0.09	1.37	0.171	−0.08
	In my opinion, brain death is not the death of a human.	2.21	1.35	2.35	1.31	0.14	−2.00	0.047	0.12
	I do not want to be confronted with topics related to death.	2.08	1.31	2.26	1.36	0.18	−2.66	0.008	0.17
	If there is a life after death, maybe I need all my organs for it.	1.40	0.75	1.49	0.77	0.09	−1.85	0.066	0.11
	*Antidonation scale*	*15.74*	*6.00*	*16.17*	*6.18*	*−0.43*	*−2.05*	*0.041*	*0.13*

^a^ *p*-values based on t-test for paired samples

In I_1_, all mean values of the **prodonation** items increased, except for the statement that many people are currently waiting for an organ and their life depends on an organ transplant. However, this might be due to a ceiling effect, because almost all participants had already agreed to this statement at t_0_. All other variables changed significantly from t_0_ to t_1_, either in I_1_, I_2_, or both. In I_1_, a significant change occurred only in the averages of the approval of the variables “An organ donation makes sense, because it gives meaning to my death” and “An organ donation makes sense, because I do not need my organs after death” (*p* = 0.001 and *p* < 0.001, respectively). In I_2_, particularly the variables related to altruistic ideas and reciprocity changed significantly, although in the opposite direction to what was expected. In the control group, the averages of the pre- and post-survey differed only marginally and not significantly (Table 2).

Some reservations and fears also changed significantly due to the intervention. In both intervention groups, agreement with the statement “I am worried that I will not really be dead when organs are removed” decreased significantly. In addition, participants agreed more strongly in the post-survey that they did not want to deal with the issue of death.

All the variables related to **antidonation** changed significantly either in I_1_, I_2_, or both intervention groups, except for the statement that organs may be needed if there is a life after death. The most pronounced effects observed were related to reductions in the fear that one might not be dead when organs are removed, that people are afraid of misuse, and that brain death is not considered to be human death. In the control group, the averages of agreement on some variables increased significantly in the context of reservations and fears in the post-survey. This might be related to further themes in the live show “Endlich!”, which deal with topics related to death (Table 3).

## Discussion

The study presented here confirms results recently published by Aust et al. [31], according to which medical cabaret both entertains the audience and stimulates critical reflection on health-related topics. It thus contributes further important insights into how to measure the effects of humor in health communication and, for the first time, it tests an innovative strategic approach by medical cabaret to promote willingness to donate organs.

Through medical cabaret, the audience could be sensitized to the issue of organ donation. The results clearly indicate that a humorous facilitation of information, specifically a humorous debunking of fears related to organ donation, may foster a multiplier effect, as almost two thirds of the intervention groups talked about this issue later on with friends and/or family. Therefore, our results are in line with a study by Campo et al. [23], in which it is stated that humorous messages have the potential to encourage recipients to exchange views with other people when a topic is presented in a humorous manner. This is important in that the intervention may thus cause a change in attitudes or behaviors not only among the recipients, but also in other people. For that reason, the coverage of medical cabaret is much broader than the audience members who are present at a live show.

The effects of this medical cabaret are clearly visible, considering the result that one third of the intervention groups who initially had a neutral or negative attitude towards organ donation changed this attitude and developed a more positive view after the intervention. The results show that the humorous messages have different effects on the individual prodonation and antidonation items. In addition to the desired positive effects on the approval ratings of the reasons for an organ donation, unintended effects also occurred. For example, the approval rating of the variable “An organ donation is meaningful, because it helps other people in need of organs” decreased significantly. These effects may possibly be explained by the reactance of respondents. Controversial health issues are often criticized and discussed. Furthermore, the intervention might also be drawing the viewer’s attention to critical aspects for the first time. Therefore, the wording used by the medical cabaret artist is of great importance. This finding confirms previous research, which describes how humor can also produce unintended effects [28].

In addition to a few unintended effects, however, the intervention yielded predominantly positive results. It significantly reduced some fears related to organ donation. The results suggest that irreversible brain function failure is more widely accepted as a diagnostic criterion for death after the live show. With regard to already-identified predictors of willingness to donate organs, this finding is promising, because the belief that brain death is not the true death of humans impacts negatively upon willingness to donate [12–14]. This has recently been shown in different groups of respondents, including teenagers [33] but also medical school students [34]. Studies consistently highlight the need to address both knowledge and psychosocial factors in order to promote willingness to donate [33–36]. Horton and Horton [37] developed a causal model of knowledge, values, and attitudes towards organ donation, towards the willingness to donate organs, and towards requesting and/or carrying an organ donor card. This model was refined into the Organ Donation Willingness Model, which claims that five factors are important for understanding and explaining willingness to donate organs: level of altruism, knowledge, attitudes, fears, and subjective norms [38, 39].

The intervention focused on all these aspects in a humorous way by using entertainment education. In this context, Miller et al. [35] emphasized the need for myth-debunking, because erroneous beliefs and irrational fears are often promoted through sensationalist media misrepresentations. For that reason, the approach of providing corrective information to address the common myths and misconceptions surrounding organ donation using medical cabaret seems to be reasonable and more promising than focusing on persuasive messages only.

One needs to keep in mind the restricted transferability of this intervention. However, a live show offered several times a week can reach a large audience in total. In addition, such an intervention might also be presented in the television, reaching even a broader audience, although one might expect differences in effect sizes between a live show and a television program.

### Limitations

This study aimed to illustrate the long-term effects of a humor-based intervention – using medical cabaret – on willingness to donate organs. It needs to be kept in mind that the audience at a live show of medical cabaret is highly selective (selection bias). Furthermore, people who are interested in the topic are more likely to participate in both the pre- and post-survey, whereas people showing fears related to organ donation may have been unwilling to answer the questions (non-response bias).

In addition, the data collected is based on subjective statements by the respondents. Therefore, a certain degree of inaccuracy might occur, which is not relevant for the psychological factors, but for the actual willingness to donate organs. It would have been desirable to set objectively measurable outcome criteria. For this, however, one would have to check whether study participants actually have an organ donor card. For this reason, when interpreting the results, it must be taken into account that the study participants may provide answers which are socially desirable or they may remember poorly or incorrectly in the follow-up interview (recall bias). In the case of socially desirable responses, the effects of the medical cabaret would in fact probably be larger, because it is expected that in the pre-survey some people will have stated that they were positive about organ donation and willing to donate organs, even though they still had reservations and fears about the topic. In addition, the post-survey was predominantly completed online. It can be assumed that the risk of socially desirable responses in an online survey is much smaller than in a paper-and-pencil questionnaire.

Furthermore, it should be noted that there are different types and understandings of humor, which may lead to diverse outcomes. Future research should take into account the variations in effects attributable to different kinds of humor.

## Conclusions

The changes in general attitudes and willingness to donate organs have shown that interventions by medical cabaret can achieve the intended persuasive effects by informing the audience about organ donation and debunking fears related to this topic. According to the results presented here, the humorous format of addressing a serious health-related topic using medical cabaret plays an important role in health communication. The persuasive effect of this humor-based format should receive a higher priority in research in order to scientifically develop further questions. In particular, the content of the humorous messages and the nature of the humor, which ultimately leads to changes in attitudes and behaviors, should be the subject of further research efforts.

## Data Availability

The data used for the current study is available from the corresponding author on reasonable request.

## Abbreviations

C: Control group
CI: Confidence interval
I: Intervention group
OR: Odds ratio
